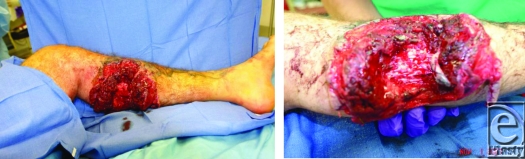# Lower Extremity Shotgun Wound

**Published:** 2011-02-25

**Authors:** William Abouhassan

**Affiliations:** Department of Plastic Surgery, Cleveland Clinic, Cleveland, OH; and Johns Hopkins Burn Center, Department of Plastic Surgery, Johns Hopkins University, Baltimore, MD

## DESCRIPTION

A 32-year-old white man presented to the emergency department after suffering a shotgun wound to the left lower extremity. He reports assault at point-blank range with a gun of unknown gauge while lying in the prone position. He was transported to the emergency department by emergency medical services personnel who started the use of peripherally inserted intravenous catheters and applied pressure to slow the bleeding.

Physical examination reveals a young, healthy appearing male in no apparent distress. Mangled gastrocnemius was clearly visible. The wound was not actively bleeding. Distal pulses (dorsalis pedis and posterior tibialis) were 2+ and the toes had good capillary refill less than 2 seconds. Motor and sensation were grossly intact.

## QUESTIONS

1. **What is the initial management of this patient?**
2. **What is essential to gather from the history?**
3. **What additional injuries are of concern, and what diagnostic tests should be ordered?**
4. **What are the principles of operative management of shotgun wounds?**
5. **What are the options for definitive closure and reconstruction?**

## DISCUSSION

This patient has sustained a shotgun wound to his left lower extremity. Shotgun wounds have a mortality rate of approximately twice that of other low-velocity weapons and the patient should undergo a thorough trauma evaluation. A secondary survey should follow, as about one-quarter of gunshot wound victims will have multiple injuries. A more complete evaluation of the affected limb must take place, including documentation of wound size, location, and associated characteristics. Initial treatment of the wound should include bedside irrigation, a clean dressing, splinting during diagnostic testing, and tetanus prophylaxis.

Three informations must be taken about the gunshot wound: activity or position at the time of the injury, type/gauge of the gun, and the distance from which the gun was used to injure the victim? The chart should include the location, dimension, and shape of the wound and any unusual features close by (ie, soot, bruising, contact wound, punctuate red marks from powder particles). Patients with shotgun wounds are at risk for vascular injury, compartment syndrome, neurologic injury, infection, and fracture. Pulses, capillary refill, color, and temperature are evaluated. Sensory testing and motor strength is evaluated. Hard signs of vascular injury include expanding hematoma, pulsatile bleeding, pulse deficit, and ischemia. However, in cases of complete arterial disruption, a weak but palpable pulse may still be present. Controversy exists regarding the need of a formal study if the vascular examination is intact, as the incidence of missed injury with physical examination alone is not greater than 1.4%. Duplex ultrasound is a quick method to evaluate questionable arterial blood flow, but it is highly operator dependant and may be difficult in the presence of tissue edema. Physical examination in conjunction with an ankle-brachial index greater than 0.90 is sufficient to rule out vascular compromise. Indications for angiography include the following: wound in close proximity to significant vascular structure, massive tissue injury, hard signs of vascular injury, detected thrill/bruit, and preexisting vascular disease. Multislice computed tomographic angiography is as sensitive for detection of arterial injury as the traditional catheter-based angiography. There should be a high index of suspicion for compartment syndrome.

Principles of operative management include early excision and debridement, ideally within the first 6 to 8 hours, as the bacterial load per gram of tissue increases 10-fold between hours 6 and 12 postinjury. Muscle should be evaluated according to the 4 Cs: color, consistency, contractility, and capacity to bleed. The wound should be reexplored at 48 to 72 hours. It is best to cleanse the wound with pulsatile lavage using copious amounts of normal saline. Any fracture should be stabilized at the time of the initial operation. Antibiotics are indicated in cases of open fracture. Early nerve exploration and repair is relatively indicated in cases with associated vascular lesion or fracture. Otherwise, it is best to delay nerve repair, as spontaneous recovery may occur.

Primary wound closure is contraindicated in shotgun wounds. The optimal time for reconstruction in these patients remains controversial. Early reconstruction (<72 hours) is favored by some because of a decreased incidence of nosocomial infection, secondary necrosis, and flap complication rate. Staged procedures (with reconstruction >72 hours) are favored when there is a need for a “second-look” operation to establish adequate debridement. The resulting tissue defect should be closed according to the principles of the reconstructive ladder: primary closure, skin graft, local flaps, and regional flaps followed by free tissue transfer.

In this patient, we chose a skin graft as the soleus muscle was still completely intact and the resulting defect did not warrant the morbidity of a flap at more than 72 hours. In cases where local flaps are the most reasonable option, the rule of thirds should be followed: for lesions of the proximal tibia a gastrocnemius flap should be used, for mid-tibia lesions a soleus flap should be used, and for distal tibia/foot/ankle lesions a free flap should be used. When a free flap is indicated, the primary goal should be to replace like with like. Cutaneous flaps that have shown consistent anatomy and a long vascular pedicle include lateral arm, radial forearm, scapular, parascapular, and anterolateral thigh flaps. Reliable muscle or musculocutaneous free flaps include latissimus dorsi, rectus abdominis, and gracilis.

## Figures and Tables

**Figure:**